# Clinical development and performance of the First to Know Syphilis Self-Test for over-the-counter usage: a *de novo* rapid test for treponemal antibody

**DOI:** 10.1128/jcm.00244-25

**Published:** 2025-08-05

**Authors:** Kevin Clark, Antony George Joyee, Diane Biddison, Sharon Rabine, Jianghong Qian, Sydney Spradlin, Vicki Thompson, Qinwei Shi, Jing Xu, Lu Zhang, Alicia Brown, Lisa Nibauer, Raji Pillai, Jody D. Berry

**Affiliations:** 1 NOWDiagnostics, Inc.Springdale, Arkansas, USA; Marquette University, Milwaukee, Wisconsin, USA

**Keywords:** syphilis, *Treponema pallidum*, diagnosis, rapid syphilis test, self-test, Over-the-Counter Syphilis Test

## Abstract

The resurgence of syphilis in the USA and globally has hastened the need for widely available syphilis testing. Early detection of syphilis is crucial for avoiding serious clinical complications and preventing the spread of infection. Self-tests enhance access to testing and promote timely treatment for positive cases. Herein, we describe the performance of First to Know Syphilis Test (FTK), the first over-the-counter (OTC) treponemal test. A prospective multi-site clinical study with 1,270 subjects was conducted, where subjects self-collected fingerstick capillary blood and self-tested without assistance. FTK test results were compared with a composite reference standard to evaluate the diagnostic performance. The FTK test exhibited an overall sensitivity of 93.4% (95% CI, 87.0% to 96.8%) and specificity of 99.5% (95% CI, 98.9% to 99.8%) and accuracy of 99%. The overall agreement was 98.9%, with Cohen's Kappa (ⱪ) value 0.93 (95% CI, 0.90 to 0.97) showing excellent agreement between the reference standard and FTK results. In addition, the test was validated in a panel of 125 clinically staged syphilis patient samples, and the results showed 100% agreement in detecting anti-treponemal antibodies in all the samples. These data show excellent performance of the FTK Test, demonstrating its utility in the screening and diagnosis of syphilis. The availability of this OTC test and its excellent performance will have a profound impact on syphilis detection and prevention strategies and could reduce comorbidities such as HIV and transmission of other STIs.

**IMPORTANCE:**

The resurgence of syphilis in the USA and globally underscores the urgent need for rapid, accessible testing. The First To Know Syphilis Test, as the first at-home, over-the-counter (OTC) test for syphilis, represents a significant breakthrough in rapid diagnostics for increased access to testing and support early diagnosis and treatment. The FTK test demonstrated excellent overall clinical performance, with 93.4% sensitivity and 99.5% specificity, confirming its reliability for syphilis detection. This novel, easy-to-use OTC syphilis test allows individuals to privately test at home and can promote testing among those hesitant or unable to seek traditional healthcare, reducing barriers like stigma and limited access. The availability of this OTC test will have a significant impact on syphilis detection and prevention strategies and reduction of comorbidities such as HIV.

## INTRODUCTION

Syphilis is a sexually transmitted infection (STI), caused by the spirochete bacterium, *Treponema pallidum*. It is transmitted by direct sexual contact or through vertical transmission from the mother to child during pregnancy and can cause substantial morbidity and mortality. The number of syphilis cases in the USA is currently on the rise. According to the recent WHO report, infection cases have escalated globally by over 1 million in 2022, reaching a total of 8 million (1), which included 700,000 cases of congenital syphilis (2). The Centers for Disease Control and Prevention (CDC) reported that the cases increased by nearly 80% between 2018 and 2022 (from 115,000 to more than 207,000) (3). The rise in syphilis can be attributed to multiple factors including social and behavioral changes in this pre-exposure prophylaxis (PrEP) era; health disparities, particularly among sexual and gender minority populations; lack of timely testing and adequate treatment; intersections with the HIV and substance use epidemics; and increased maternal transmission leading to congenital syphilis infections (4). Syphilis is a chronic, multi-stage disease. The natural course of syphilis progresses through four stages: primary, secondary, latent, and tertiary stages, with the primary and secondary stages being the most infectious. It is optimal to treat syphilis in its early stages for favorable patient outcomes; however, cure in later stages may require additional or differing penicillin regimens. If left untreated, syphilis can lead to serious complications and permanent damage in the nervous and cardiovascular systems, which can be life-threatening (5–7). Syphilis during pregnancy, if untreated or treated late, can result in serious adverse birth outcomes including stillbirths, neonatal deaths, prematurity, low birth weight, and congenitally infected infants (8, 9). As many people are often asymptomatic or may not notice symptoms (10), screening is a crucial step in the diagnosis. There is no natural immunity to syphilis, and the antibodies generated from past infection are insufficient to confer protection against re-infection (11, 12).

The laboratory diagnosis of syphilis is complex and necessitates a combination of clinical and laboratory criteria to differentiate current or past infection and absence of infection (13, 14). Culture for *T. pallidum* is cumbersome and is available only in selected research laboratories, and there is no Food and Drug Administration (FDA)-cleared PCR test for syphilis at present. Darkfield microscopy is a direct detection test for *T. pallidum* that can diagnose early primary syphilis (sensitivity 75% to 100%); however, it requires samples from moist lesions or exudates, along with highly trained technicians and specialized equipment. It is not recommended for secondary syphilis due to low sensitivity (4). Serological testing remains the mainstay for diagnosing syphilis infection. Serology is divided into two different types of tests: non-treponemal tests (NTTs), such as the rapid plasma regain [RPR] test and the Venereal Disease Research Laboratory (VDRL), and treponemal tests (TTs), such as the *T. pallidum* particle agglutination assay (TP-PA) and Fluorescent Treponemal Antibody Absorption (FTA-ABS). While TT is often positive for life, NTT titers usually decrease after treatment. However, in certain cases, especially in late stages of syphilis, non-treponemal antibodies may persist at low levels for extended periods. The traditional NTTs are cost-effective and useful for initial screening and monitoring of syphilis treatment; however, they can be affected by false-positive and false-negative results. TTs provide higher specificity and sensitivity for confirming syphilis but cannot effectively monitor the disease activity after treatment or differentiate between active and past infections. Thus, both TTs and NTTs have inherent limitations due to variability in their diagnostic performance, hampering syphilis control and prevention efforts in real-world settings (9). A single serologic NTT or TT is not sufficient for diagnosis, and therefore a combination of positive TT and reactive NTT is required for the diagnosis of syphilis infection. In the past, syphilis screening mainly followed a traditional testing algorithm, starting with NTT, and the positive results required further confirmation through more specific TTs (9, 13). With increased availability of various treponemal-specific immunoassays including enzyme immunoassays (EIAs) and chemiluminescence immunoassays (CIAs), recently, many laboratories are increasingly adopting a “reverse sequence screening” algorithm, which begins the screening with a TT, followed by confirmation of the reactivity with an NTT. When the NTT is non-reactive, a second but different treponemal test is performed to determine if the first treponemal test was a false positive (15). Regardless of which algorithm is used, it is important to consider the sensitivity and specificity of these assays in clinically characterized sera, stratified by the stage of syphilis (16), and all screening results must be correlated with clinical diagnosis, which includes patients’ symptoms, previous history, and sexual risk factors to make an accurate diagnosis (17).

Timely testing for syphilis is critical for the prompt diagnosis of infections, enabling effective clinical management and preventing disease transmission (7). Expanding access to testing facilitates increased detection rates and timely intervention. Various testing methods are now available, each differing in convenience, speed, and level of oversight. At-home tests involve self-collection of samples sent to a reference lab for analysis. Some tests allow self-collection at a provider’s office, enabling patients to independently collect samples while the testing is performed by the provider. Point-of-care (POC) tests are conducted on-site by healthcare providers, delivering rapid results during the visit. Over-the-counter (OTC) tests enable individuals to self-collect samples and perform tests in the privacy of their home, providing an accessible option for timely detection, which could facilitate prompt early diagnosis and treatment. Rapid OTC tests have become entrenched in the society since the COVID-19 pandemic with broad acceptance of being able to self-test at home. These tests are easy to perform and inexpensive, so repeated testing is practical, and test results are available immediately. They are useful for quick screening for syphilis and initiating treatment, especially in settings where access to traditional healthcare is limited. Moreover, the convenience of at-home self-testing is significantly beneficial, particularly for underserved minority populations who often face challenges in accessing STI care, such as appointment difficulties, especially if uninsured, and the stigma associated with STIs, which can lead to decreased engagement in sexual health services. Thus, easy-to-access rapid OTC tests could reduce barriers to syphilis detection. It is important to note that, while rapid treponemal-specific antibody tests for syphilis inform whether an individual has had a past or current infection, they cannot determine active infection. Therefore, it is important to confirm these findings through consultation with regional health officials and patient examinations.

The First To Know Syphilis Test (NOWDiagnostics Inc.) is the first at-home, over-the-counter (OTC) treponemal test for syphilis. It is a novel buffer-less lateral flow immunoassay rapid test device designed for rapid detection of *T. pallidum* antibodies in human whole blood (capillary) from individuals suspected of having a syphilis infection. The test received FDA marketing authorization in August 2024 under a *de novo* classification pathway for novel, low- to moderate-risk devices without existing predicates. Here, we report the findings from a clinical study evaluating the performance of the First To Know Syphilis Test.

## MATERIALS AND METHODS

**Fig 1:**
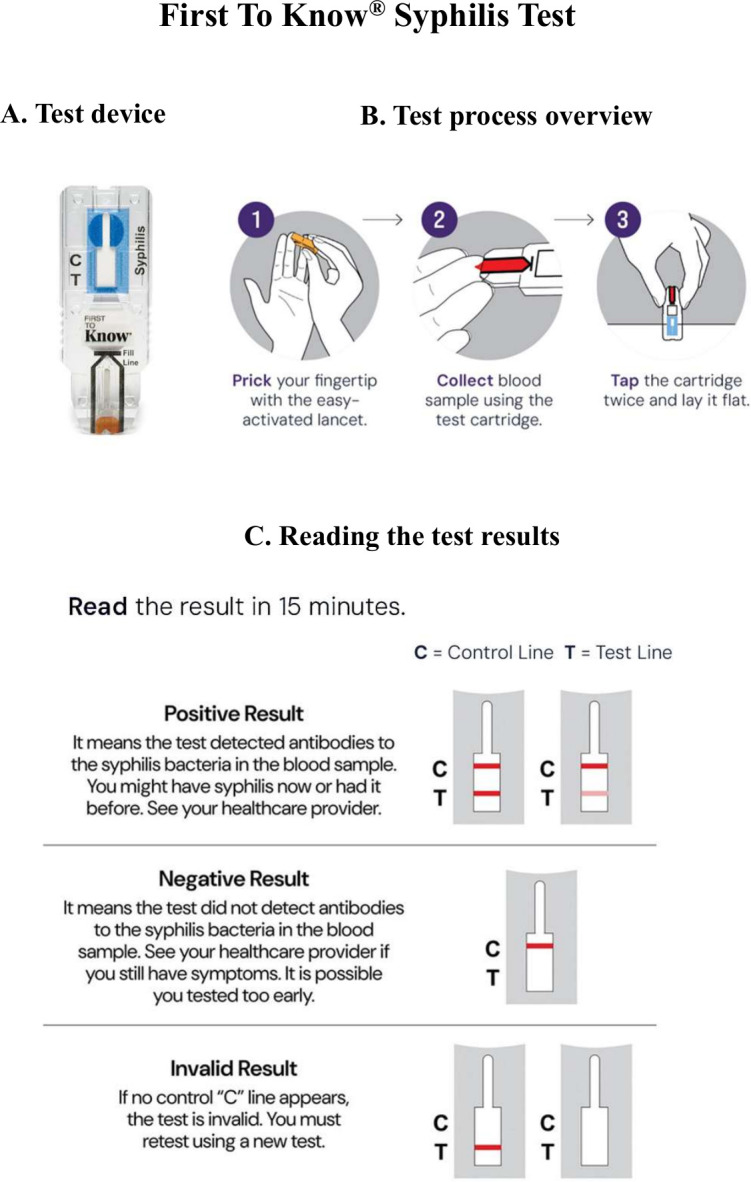
First To Know Syphilis Test device, test process, visual reading, and interpretation of test results This figure depicts the components of the First To Know Syphilis Test device, outlines the testing procedure, and illustrates how to interpret the visual results.

The First To Know Syphilis Test is a qualitative rapid membrane immunochromatographic assay for the detection of *T. pallidum* antibodies in human whole blood (capillary). It is a simple buffer-less lateral flow visually read test device and requires a fingerstick and whole blood to run the test in as little as 15 minutes. There is no need for a swab nor a stand to run this test. Briefly, a drop (40 µL) of human capillary whole blood is added to the test device cassette, where blood is drawn into the fill zone. As the sample flows into the porous test strip, syphilis-specific antibodies in the sample bind at the test band location. The appearance of a visible test band indicates the sample contains a detectable level of the anti-treponemal antibody. The internal control and the appearance of the control band line assure that the sample is applied correctly, and the test components are working properly. The test is a chromatographic immunoassay that delivers results as early as 15 minutes, but it must be read within 30 minutes to be considered valid. To ensure accurate results, users should adhere to the Instructions for Use (IFU) (the IFU, FAQs, test limitations, and other resources related to the test can be found at: https://nowdx.com/). The stability of the test device is up to 18 months when stored within the specified temperature range of 15°C to 30°C. The First To Know Syphilis Test device, test process overview, visual reading, and interpretation of test results are depicted in Fig. 1.

### Study subjects, sample collection, and syphilis testing

To evaluate the performance of the First To Know Syphilis Test, an IRB-approved prospective clinical study was conducted in six geographically diverse sites across the United States. These sites comprised clinical research centers and community health organizations (Tempe, AZ; Las Vegas, NV; Los Angeles, CA; Spokane, WA; West Orange, NJ and North Miami, FL). The sample size for the test performance evaluation was determined based on the FDA guidance documents. Individuals who met the inclusion criteria—sexually active persons aged 18–64, including those who have previously been diagnosed with or tested positive for syphilis—were consecutively enrolled in the study. Exclusion criteria for both cohorts included individuals under 18, over 64, or with limited or no reading skills. For the clinical study, subjects were enrolled from 1 September 2021 through 17 October 2023 from two cohorts: one cohort of sexually active individuals (18–64 years old) and another cohort of expectant mothers ( ≥18 years old). The enrolled subjects self-collected capillary blood from a fingerstick and performed self-testing according to the instructions for use with the First To Know Syphilis Test. The test was conducted by participants in a private, home-like setting created at the study sites, without any assistance in performing the test or interpreting the results. Clinical staff members did not review the OTC test. Upon completion of the testing, each participant filled out a Case Report Form (CRF) on paper. The de-identified data were submitted to the study team by the site operator, who was blinded to the participants’ test results. Additionally, the participants completed a post-study questionnaire to capture their user experience and evaluate their ability to execute the tests and interpret the results based solely on the Instructions for Use (IFU) and the ease of use of the test. The clinical study protocol and the informed consent form for all the study sites were approved by Advarra IRB, and the same protocol was adhered to without deviations. If the test results were incomplete, invalid, or not performed or lab results were missing for any samples or could not be obtained due to insufficient material for testing, they were excluded from the analysis. There were no adverse events in the performance study.

### Composite reference result

Venous whole-blood samples were also collected from the subjects during the same visit as for the OTC test. Plasma and serum samples prepared from whole blood were sent and tested by a reference lab using FDA-cleared comparator tests. The results of the First To Know Syphilis Test were compared to comparator test results derived from an algorithm that includes both treponemal and non-treponemal tests, as stipulated by the FDA. The comparator tests included three different assays: two treponemal tests (BioPlex 2200 Syphilis Total and SERODIA- TPPA Test) and one non-treponemal test (Wampole Impact RPR Test). Serum was used for the RPR and TPPA tests, whereas plasma was used for BioPlex testing. The reference of “true positive” for syphilis test positivity was established if at least two of the three tests were positive, and this composite reference standard was used for comparison with the First To Know Syphilis Test to evaluate the test sensitivity and specificity. The participants did not have reference standard test results at the time of self-testing with the First To Know Syphilis Test. The Standards for Reporting of Diagnostic Accuracy Studies (STARD) reporting guidelines were followed to ensure the completeness and transparency of reporting diagnostic accuracy studies (18).

### Clinically staged syphilis patient samples and testing with First To Know Syphilis Test

In addition to the clinical study evaluation, we validated the diagnostic accuracy of the First To Know Syphilis Test in a panel of characterized serum samples of known positive clinically staged patients obtained from the CDC. The panel included serum samples from patients with primary syphilis (*n* = 25), secondary syphilis (*n* = 56), early latent syphilis (*n* = 16), and late latent syphilis (*n* = 28).

### Statistical analysis

Statistical analysis was performed using GraphPad Prism 9 (GraphPad Software, Inc.) The performance of the First To Know Syphilis Test was evaluated based on comparison with the composite reference standard using a dichotomous approach, two-by-two crosstab analysis for the calculation of sensitivity (positive percent agreement [PPA]), specificity (negative percent agreement [NPA]), positive predictive value (PPV), and negative predictive value (NPV), and accuracy. The concordance analysis for diagnostic performance was determined using the Cohen’s kappa (κ) value according to the criteria proposed by Landis & Koch; e.g., values more than 0.8 were considered “almost perfect agreement” (19).

## RESULTS

**Fig 2:**
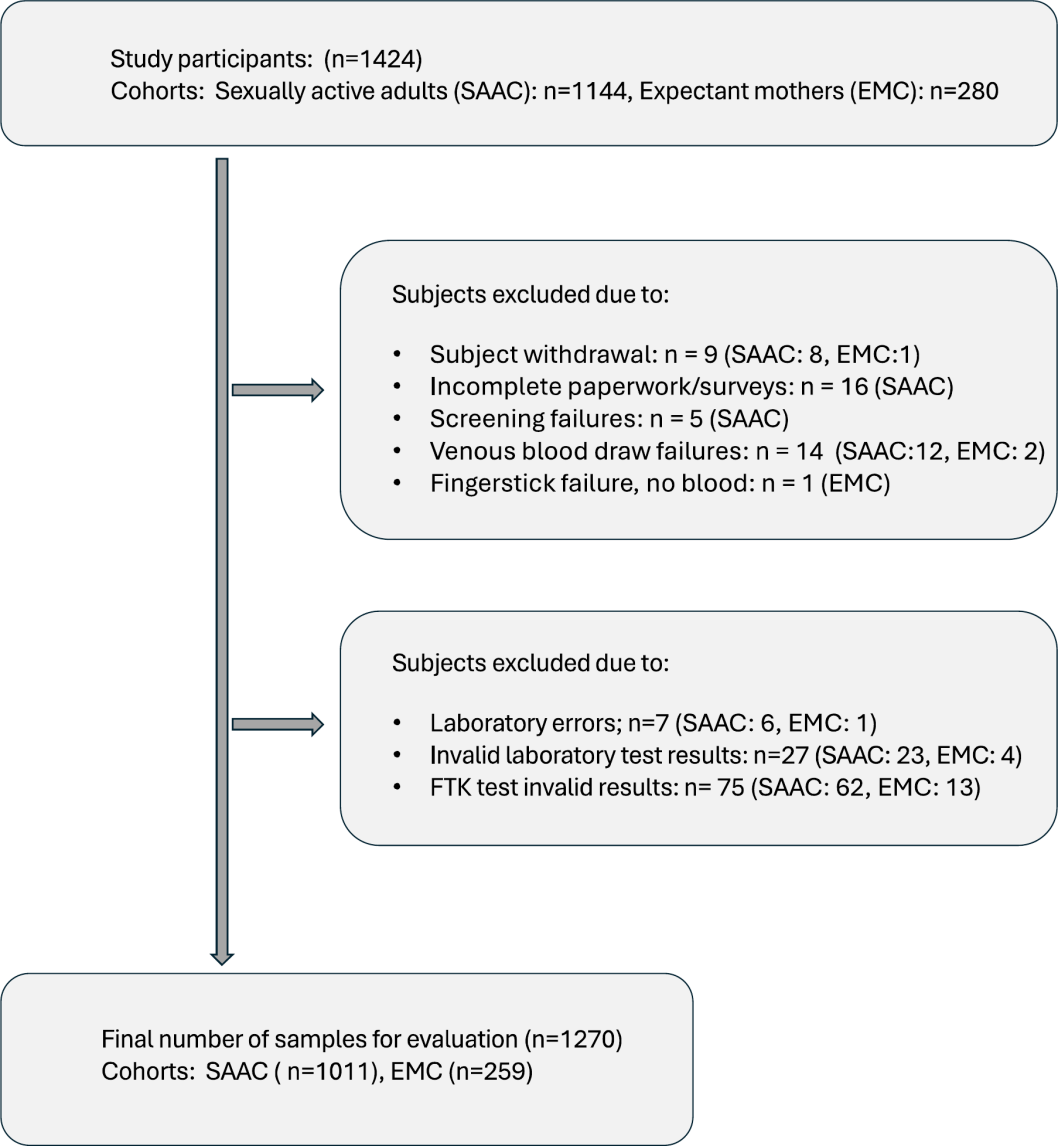
Flowchart of study participants. The flowchart of study participants illustrates the enrollment, exclusions, and final inclusion of study subjects for the evaluation of the First To Know Syphilis Test. SAAC; sexually active adult cohort, EMC; expectant mother cohort, FTK; First To Know Syphilis Test.

 Of 1,424 prospectively enrolled subjects, 154 subjects were excluded due to incomplete paperwork/surveys (*n* = 16), subject withdrawal (*n* = 9), screening failures (*n* = 5), venous blood draw failures (*n* = 14), fingerstick failure (*n* = 1), laboratory errors (*n* = 7), invalid comparator test results (*n* = 27), and subject test device invalid results (*n* = 75) due to the lack of the control line in the First To Know test device following the test. A total of 1,270 study subjects (comprising 1,011 samples from the sexually active adult cohort and 259 samples from the expectant mother cohort, combined for the final evaluation) who obtained valid test results for First To Know test and laboratory comparator tests were included to evaluate the diagnostic performance of the First To Know Syphilis Test (see Fig. 2, flow chart for study participants for details). The overall positivity for syphilis in this study was determined to be 8.3% (106/1,270) based on the composite reference standard. The First To Know test showed positivity in 99 out of the 106 positive samples. Among the 1,164 negative samples, 1,158 samples were negative by the First To Know test. Based on the composite reference, the First To Know test yielded an overall sensitivity of 93.4% (95% CI, 87.0%–96.8%) and specificity of 99.5% (95% CI, 98.9%–99.8%) (Table 1). The test demonstrated an accuracy of 99% in this evaluation. The overall agreement between the reference standard and the First To Know test was 98.9%, with Cohen’s Kappa (ⱪ) value 0.93 (95% CI, 0.90–0.97) showing within the scale of “almost perfect agreement” (Kappa between 0.81 and 1.00).

**TABLE 1:** Evaluation of performance characteristics of the First To Know Syphilis Test compared to the composite reference, as required by the FDA for OTC^*a*^

First To Know assay	Reference (composite)		
	Positive	Negative	Subtotal
Positive	99	6	105
Negative	7	1,158	1,165
Subtotal	106	1,164	1,270
Sensitivity/PPA	93.4% (95% CI, 87.0%–96.8%)		
Specificity/NPA	99.5% (95% CI, 98.9%–99.8%)		
Positive Predictive Value (PPV)	94.3% (95% CI, 88.1%–97.4%)		
Negative Predictive Value (NPV)	99.4% (95% CI, 98.8%–99.7 %)		
Accuracy	99.0% (95% CI, 98.3%–99.5%)		

^*a*^ For reference standard, positive and negative results were determined by a composite algorithm, as described in the Methods.

With respect to the two study cohorts, the syphilis test positivity determined by the composite reference standard in the sexually active adult cohort group was 9.7% (98/1,011), whereas in the pregnant women cohort, a lower syphilis positivity rate of 3.1% (8/259) was observed. The First To Know Syphilis Test positivity rates were 9.2% (93/1,011) and 2.3% (6/259), respectively, in the sexually active adult and pregnant women cohorts. The sensitivity and specificity of the First To Know Syphilis Test for the sexually active adult cohort group were 94.9% (95% CI, 88.5%–98.3%) and 99.6% (95% CI, 98.9%–99.9%), respectively, while in the pregnant women group, the sensitivity and specificity of the test were 75.0% (95% CI, 40.9%–92.9%) and 99.2% (95% CI, 97.1%–99.8%), respectively (shown in Table 2).

**TABLE 2:** Study cohorts and test performance

Study cohorts Total (*n* = 1,270)	First To Know Syphilis Test	
	Sensitivity/PPA Test +/Ref + (%, 95% CI)	Specificity/NPA Test −/Ref − (%, 95% CI)
Sexually active cohort (*n* = 1,011)	93/98 (94.9%, 95% CI, 88.6%–97.8%)	909/913 (99.6%, 95% CI, 98.9%–99.8%)
Pregnant cohort (*n* = 259)	6/8 (75.0%, 95% CI, 40.9%–92.9%)	249/251 (99.2%, 95% CI, 97.1%–99.8%)

The overall test results for the various laboratory comparator tests and The First to Know Syphilis test are summarized in Table S1. Among the 106 true positives determined by the composite reference standard, 99 tested positive with the First To Know Syphilis Test. Of the 41 participants who tested positive on all three reference tests (Bioplex +, RPR +, TPPA +), 40 (97.6%) tested positive with the First To Know test (Table 3). Additionally, of the 64 participants who were positive for both treponemal tests (Bioplex and TPPA) but negative for RPR, 59 (92.2%) tested positive with the First To Know test (Table 3). There was no significant difference in First To Know test positivity between those positive by both treponemal and non-treponemal tests (40/42, 95.2%) and those positive only on the two treponemal tests (59/64, 92.2%). The seven false negatives included five positives by both Bioplex and TPPA, one positive across all laboratory comparator tests and one positive by TPPA and RPR. We speculate that the false negatives could be due to low antibody levels in early or low-level infections. The IFU recommends that individuals who test negative but still suspect infection should retest after 10 days after symptom onset or consult a healthcare provider for further evaluation. Additionally, differences in specimen types, such as purified serum or plasma used for the laboratory comparator tests versus whole blood specimens tested by the First To Know test, could also have contributed to missed detections. Overall, the First To Know test demonstrated strong overall concordance with the reference standard across various test result combinations (Table 3).

**TABLE 3:** Test result combinations for true positives vs First To Know Syphilis Test

Comparator test result combinations	Composite reference no. positive	First To Know Syphilis Test	
		Pos^*a*^	Neg^*b*^
Bioplex + RPR + TPPA+	41	40	1
Bioplex + RPR + TPPA−	0	0	0
Bioplex− RPR +TPPA +	1	0	1
Bioplex + RPR− TPPA+	64	59	5
Total	106	99	7

^*a*^ Pos; Positive.

^*b*^ Neg; Negative.

The two laboratory treponemal assays, Bioplex and TPPA, used in the reference algorithm showed a high concordance of 97.6% (kappa, 0.86; 95% CI: 0.81–0.91) across all the samples tested. There were 30 discordant results, including 16 samples that were positive by Bioplex but negative by TPPA and 14 samples that were negative by Bioplex but positive by TPPA (Table S2; Supplemental information). The First To Know Syphilis Test demonstrated a consistent and high level of agreement with both the Bioplex and TPPA treponemal tests. Comparison of the First To Know Test with each of these tests showed an overall agreement of 98.1% and a Cohen’s kappa value of 0.88 (95% CI: 0.84–0.93), indicating almost perfect agreement (Table 4) in both the comparisons. These data highlight the robustness and reliability of the First To Know Test and suggest that it can perform comparably to established laboratory treponemal assays, supporting its potential utility in clinical and diagnostic settings.

**TABLE 4:** Comparison of First to know Syphilis Test with laboratory treponemal tests

Tests	Bioplex treponemal test		Total	Agreement (%)	K value (95% CI)
First To Know Test	Positive	Negative			
Positive	101	4	105	98.1%	0.88 (0.84 to 0.93)
Negative	20	1,145	1,165		
Total	121	1,149	1,270		

	TPPA		Total	Agreement (%)	K value (95% CI)
First To Know Test	Positive	Negative			
Positive	100	5	105	98.1%	0.88 (0.84 to 0.93)
Negative	19	1,146	1,165		
Total	119	1,151	1,270		

RPR was positive in 49 of the 1,270 subjects. Among these RPR-positive samples, the First To Know Test showed positivity in 40 cases. There were 65 samples that were positive by First To Know test but negative by RPR and 9 samples that were negative by First To Know test but positive by RPR. The overall agreement between the two tests was 94.2%, with a kappa value of 0.49 (95% CI: 0.40–0.59), indicating moderate agreement. These data reflect the inherent differences between treponemal and non-treponemal tests, where the First To Know test detects treponemal antibodies indicative of exposure to *T. pallidum*, whereas RPR detects non-treponemal antibodies that can be associated with active infection but are not specific to syphilis and can be present in other conditions. Further, the sensitivity of the First To Know test was analyzed across different RPR titers. We observed a clear trend in sensitivity and specificity relative to increasing RPR titers. Notably, as RPR titers increased, the sensitivity of the First To Know test showed an upward trend, reaching 100% at titers of 1:8 and above, although with wider confidence intervals due to fewer positive samples at higher titers. The specificity remained consistently high, above 90%, across all RPR titer levels (summarized in Table S3). Together, these data suggest that the First To Know test performs reliably across a range of RPR titers, with increased sensitivity at higher titers that are more associated with active infection.

In addition to the clinical performance study, we also evaluated the First To Know Syphilis Test in a set of 125 characterized serum samples from known positive clinically staged patients. The First To Know Syphilis Test showed 100% sensitivity in detecting anti-treponemal antibodies in all the samples tested, having absolute agreement with the reference standard. The breakdown of different clinically staged groups and test positivity are shown in Table 5.

**TABLE 5:** First To Know Syphilis testing in clinically staged syphilis patients (*n* = 125)

Clinical stage of syphilis Samples (*n* = 125)	Number tested	Test positivity number (%)
Primary syphilis	25	25 (100%)
Secondary syphilis	56	56 (100%)
Early latent syphilis	16	16 (100%)
Late latent syphilis	28	28 (100%)

## DISCUSSION

Syphilis is a preventable and curable bacterial sexually transmitted infection, but if left undiagnosed and untreated, it can result in serious downstream health outcomes. Further, there is a higher risk of acquiring HIV and other STIs (20, 21). Therefore, rapid and accurate detection of syphilis is indispensable to ensure treatment and to control the transmission of the disease. In the present study, we evaluated the diagnostic performance of the First To Know Syphilis Test in a large real-world lateral flow study. The findings of the present evaluation based on self-testing by the study subjects showed excellent overall clinical performance of the First To Know Test (93.4 .% sensitivity, 99.5% specificity, and 99% accuracy) compared with the reference method. This also meets the WHO-recommended criteria of a minimum of 85% sensitivity and 95% specificity for syphilis rapid tests (22). The sensitivities of commercially available POC tests are in the range of 76%–98%, with 90% to 99% specificities in varied settings (9, 23–27). In addition to the clinical performance study, we validated the diagnostic accuracy of the First To Know Test in a panel of characterized samples from clinically staged syphilis patients. Our results showed that the First To Know Test showed 100% sensitivity in detecting anti-treponemal antibodies in all the tested samples in the panel, demonstrating diagnostic efficacy. These results suggest that the First To Know Test has the potential to aid accurate syphilis diagnosis in high-risk patient populations with specific clinical stages; however, larger and more detailed studies are needed to confirm the present finding.

  Concurrent positivity in both RPR and treponemal tests is typically associated with active syphilis infections. In the present study, notably, the First To Know Syphilis Test was positive in 95.2% of the cases that were positive by both treponemal and RPR tests. This finding suggests its strong potential to detect most of the active infections, although as a treponemal test, it cannot distinguish between active and past infections on its own, and the diagnosis requires a combination of treponemal and non-treponemal tests. It has been shown that the sensitivity of treponemal tests increases with higher RPR titers (28, 29). Consistently, analysis of the First To Know Test in relation to RPR titers revealed that the test sensitivity increased with higher RPR titers, reaching 100% at titers of 1:8 and above. These findings suggest that the First To Know Test is effective across a broad range of RPR titers, with particularly enhanced sensitivity in cases more likely to reflect infectious syphilis. These results, coupled with the data demonstrating its ability to detect all known positive samples from clinically staged syphilis patients, highlight the potential of this OTC test as a rapid screening tool capable of detecting most active infections and different disease stages across various clinical and community settings. This is consistent with the test’s design to detect all antibody types, treponemal IgM, IgG, and IgA, which vary in their temporal expression during infection. While these findings are promising, further studies are needed to comprehensively evaluate its performance specifically in active syphilis cases.

Our findings also indicated that the First To Know Syphilis Test can perform comparably to the established laboratory treponemal assays. Comparing the First To Know test with each of the reference treponemal tests, Bioplex and TPPA, revealed identical levels of concordance and near-perfect agreement (kappa = 0.88; Table 4) in both comparisons. When taking treponemal test positivity as the reference (considering positivity when both the laboratory-based treponemal tests are positive), the sensitivity of the First To Know Test slightly increased to 94.3%, while its specificity remained at 99.5% (not shown), relative to the evaluation using the composite reference standard (which showed a sensitivity of 93.4% and specificity of 99.5%). These findings underscore the consistent performance and reliability of the First To Know Syphilis test. Taken together, the excellent performance and high agreement with laboratory-based treponemal assays suggest that this OTC test can be included in syphilis testing algorithms, though further assessment in diverse settings is recommended.

The current resurgence of syphilis in the United States and the alarming spread of infections underscore the critical need for rapid diagnosis and containment (2, 3, 30). The overall positivity rate of 8.3% in this study population indicates a high seroprevalence of syphilis. This underscores the critical importance of accessible, self-administered testing options for populations with potentially elevated risk, even within broad community settings. Currently, syphilis incidence is increasing more rapidly among men who have sex with men (MSM) than any other U.S. subpopulation (31, 32). Consistent with literature, we observed that syphilis test positivity in MSM was significantly higher than that of non-MSM (32.8% vs 6.9%, p <0.001) (data not shown). In addition, there has been a dramatic increase in congenital syphilis over the 4-year period from 2018 to 2022 (3), which clearly necessitates early detection. Increasing the coverage and frequency of syphilis testing are crucial elements of any control effort to allow earlier detection and treatment, interrupting transmission, which aids in controlling the spread of syphilis. Specifically, focusing testing on high-risk populations like MSM, female sex workers, and routine syphilis testing during pregnancy can significantly impact syphilis control. The *de novo* FDA marketing authorization and consequent availability of the First To Know Syphilis Test as the first syphilis OTC test represent a key advancement in the landscape of rapid diagnostics for syphilis, which would enable increased access to testing and early diagnosis and will support the universal effort in preventing the transmission of infections. While POC tests were available prior to the First To Know marketing authorization, currently, the First To Know Syphilis Test is the only at-home, OTC test for syphilis, which facilitates self-testing. It should be emphasized that positive test results from this test should be followed by additional laboratory testing through a healthcare provider to confirm the diagnosis. Nevertheless, it serves best as a screening tool for high-risk individuals who may have an identifiable infection, if they have never had syphilis or been treated for syphilis in the past. It should be noted that, as a treponemal test, the First to Know Syphilis Test is expected to be positive for individuals previously diagnosed with syphilis, even if they were successfully treated. The test cannot determine whether there has been a re-infection with syphilis. According to the surveillance definition, the incubation period after exposure is considered up to 90 days; therefore, a negative result does not indicate the patient does not have syphilis. Testing should be repeated in 6–8 weeks, especially for those at high risk. This is a limitation inherent in all syphilis serology tests. The First To Know Test has the potential to substantially enhance testing uptake and alleviate stigma associated with syphilis, thereby facilitating access to care for individuals at risk. However, the OTC test should not be viewed as a substitute for comprehensive diagnostic practices, and it is essential that results are evaluated in accordance with healthcare guidelines for accurate diagnosis and effective management of syphilis. Importantly, this is not a direct pathogen test; interpreting both positive and negative results is complex and influenced by factors such as delay in seroconversion, uncertainty regarding past and present infection status, and the need for a comprehensive physical examination and health department validation, all of which must be conducted by a qualified healthcare provider.

The availability of an over-the-counter test with at-home results like the First To Know Syphilis Test has important implications that extend beyond testing for convenience. By being readily available, it could increase the initial volume of screening, which is the first step toward diagnosing syphilis, which is crucial for early treatment and prevention of serious complications. Home-based self-testing for STIs such as syphilis provides several benefits over conventional clinic/laboratory-based testing. In addition to the simplicity in performing the test, it provides privacy, without fear of judgment, and therefore offers significant advantages over currently available POC tests typically used in healthcare settings by trained healthcare professionals. Self-testing at home can be a beneficial strategy to reach more at‐risk individuals who are reluctant or otherwise may never seek testing. One of the major impacts of self-testing for STIs/HIV is in increasing access to marginalized or disadvantaged populations due to challenges such as stigma, geographic distance to clinics that offer testing, and privacy and confidentiality concerns. In fact, it has been shown to be effective in the case of HIV, enhancing uptake and frequency of testing to reduce barriers for those at high risk who otherwise rarely get tested (33, 34). The recent CDC randomized clinical trial called Evaluation of Rapid HIV Self-testing Among MSM Project (eSTAMP) study showed that self-testing increased the uptake and frequency in testing and resulted in people seeking care or additional testing after obtaining a positive result (34). These programs and other studies in the United States exemplify the high level of acceptability and effectiveness of home testing for STIs. With the availability of an OTC test like the First To Know Syphilis test, it is feasible to implement similar programs for syphilis, which could help control the spread of infection. In this regard, the First To Know Syphilis Test’s ease of use with a simple fingerstick blood micro sampling (there was only one fingerstick failure, whereas more collection failures were observed for venous blood collection; See Fig. 2) and short time to obtain results with high sensitivity and specificity can be very advantageous for self-testing and to facilitate early diagnosis. Our data showed that the First To Know test is robust for self-testing in different settings; the usability studies (refer to supplemental information for details on usability studies) demonstrated that the error rate during self-testing with First To Know was very minimal, underscoring the ease of use and suitability for testing by lay users.

There are some limitations to our study; the study population largely represents a community setting, and the performance in high-risk groups was not evaluated. Consequently, the results from the current study might not reflect its performance in high-risk populations, thus possibly underestimating the test performance. Therefore, it is important to conduct evaluations of the First To Know Syphilis Test in various high-risk populations including vulnerable and underserved populations as this is valuable for assessing how well this self-test functions in conditions of higher disease prevalence and will provide a comprehensive understanding of the test’s effectiveness and its implications for the diagnosis and treatment of syphilis. In our study, individuals with a history of past STI or treatment were not excluded, and it is possible that some of the positive subjects may have had syphilis previously. Given that the First To Know Test is a treponemal test that detects both past and present infections, the results may not accurately reflect the prevalence of active syphilis infections. Nevertheless, this inclusion broadens the study’s relevance as it reflects the real-world scenario, where many individuals may have had past syphilis infections or treatment. The observed sensitivity of the test in pregnant women in our study was 75% (95% CI: 40.9%–92.9%), detecting 6 out of 8 positive cases with two false negatives (Table 2). However, the small sample size and low positivity rate, combined with the wide confidence interval, limit our ability to draw definitive conclusions about the test’s performance in this group. Considering the significance of syphilis testing in pregnant women, it is important to have further detailed clinical studies in pregnant women with large sample sizes. In addition, future research involving larger, clinically stratified patient populations will be important to strengthen our findings.

In conclusion, the present study observations showed an excellent overall diagnostic performance of the First To Know Syphilis Test demonstrating its utility as a valuable tool in the screening and diagnosis of syphilis. As the incidence of syphilis continues to rise in the United States and worldwide, the availability of this OTC test can have a profound impact on syphilis detection and prevention strategies and could support reduction of co-morbidities such as HIV and other STI transmission.

## Data Availability

All data supporting the findings of this research study are described in the manuscript and relevant details are available upon reasonable request. Due to the involvement of human subjects and nature of the STI study, the data sets are not shared in public repositories due to the limitations on ethical and privacy concerns.

## SUPPLEMENTAL MATERIAL

Supplemental material jcm.00244-25-s0001.docx Tables S1 to S3, and Usability studies.

ASM does not own the copyrights to Supplemental Material that may be linked to, or accessed through, an article. The authors have granted ASM a non-exclusive, world-wide license to publish the Supplemental Material files. Please contact the corresponding author directly for reuse.
