# Supplementary material for: Clinical development and performance of the First to Know Syphilis Self-Test for over-the-counter usage: a de novo rapid test for treponemal antibody
Source: J Clin Microbiol. 2025 Aug 5;63(9):e00244-25. doi: 10.1128/jcm.00244-25 (PMC12421861; doi:10.1128/jcm.00244-25)
Supplement: Supplemental material — Tables S1 to S3, and Usability studies. [file jcm.00244-25-s0001.docx]

**Supplemental Information**

**Table S1. Overall test results for laboratory comparator tests**

**and First to Know® Syphilis Test (n=1270)**

| **Test Results** | **Bioplex**  **(Treponemal)** | **RPR**  **(Non-treponemal)** | **Serodia- TP-PA**  **(Treponemal )** | **First To Know® (Treponemal OTC)** |
| --- | --- | --- | --- | --- |
| Pos | 121 | 49 | 119 | 105 |
| Neg | 1149 | 1221 | 1151 | 1165 |

Pos; Positive, Neg; Negative

**Table S2: Comparison of the laboratory treponemal tests**

| **Tests** | **Bioplex**  **treponemal test** | | **Total** | **Agreement ( %)** | **K value**  **(95% CI)** |
| --- | --- | --- | --- | --- | --- |
| **TPPA** | Positive | Negative |  |  |  |
| Positive | 105 | 14 | 119 | 97.6% | 0.86 (0.81-0.91) |
| Negative | 16 | 1135 | 1151 |  |  |
| Total | 121 | 1149 | 1270 |  |  |

**Table S3: Analysis of First To Know® Syphilis Test positivity vs. RPR titers**

|  | **RPR Results** | | **First To Know^®^ Syphilis test** | |
| --- | --- | --- | --- | --- |
| **RPR titer** | **Reactive** | **Non-reactive** | **Sensitivity**  **(%, 95% CI)** | **Specificity**  **(%, 95% CI)** |
| 1:1 | 49 | 1221 | 81.6%, 68.0 - 91.2% | 94.7%, 93.2 - 95.9% |
| 1:2 | 32 | 1238 | 75.0%, 56.6 - 88.5% | 93.5%, 91.9 - 94.8% |
| 1:4 | 18 | 1252 | 88.9%, 65.3 - 98.6% | 92.9%, 91.3 - 94.3% |
| 1:8 | 11 | 1259 | 100.0%, 71.5 - 100.0% | 92.5%, 90.9 - 93.9% |
| 1:16 | 6 | 1264 | 100.0%, 54.1 -100.0% | 92.2%, 90.6 - 93.6% |
| 1:32 | 4 | 1266 | 100.0%, 39.8 - 100.0% | 92.0%, 90.4 - 93.5% |
| 1:64 | 2 | 1268 | 100.0%, 15.8 - 100.0% | 91.9%, 90.2 - 93.3% |

**Usability studies**

First To Know® Syphilis Test usability was assessed in two studies. For the first, all 1345 participants enrolled in clinical study were observed while performing testing and any difficulties were noted. A second study enrolled 20 participants to assess lay users' execution of the First To Know Syphilis test workflow using the written instructions alone. Following both studies, a questionnaire was issued to participants to assess ease of use. The results of both studies demonstrated that the First To Know Syphilis test is easy to use by lay users. User label comprehension was assessed using two sets of questionnaires. One questionnaire was issued to all 1345 participants following the original clinical study. A second questionnaire was issued to 40 participants in a supplemental label comprehension study (20 participants in the supplemental usability study also participated in the supplemental label comprehension study). Both questionnaires were aimed at evaluating users' understanding of key communication messages (e.g., FAQ messages and warnings) found in the labeling. The results of user label comprehension demonstrated that the First To Know Syphilis Test labeling is easy to understand by lay users.
